# A proposed anxiety–hypertonic pelvic floor axis in postpartum women: a narrative review of mechanistic links and rehabilitation implications

**DOI:** 10.3389/fsurg.2026.1929266

**Published:** 2026-09-24

**Authors:** Fenghua Chen, Yi Chen, Yiping Huang

**Affiliations:** 1 Department of Obstetrics and Gynecology, Puyan Street Community Health Service Center, Hangzhou, Zhejiang, China; 2 Third Clinical Medical College School of Rehabilitation Medicine, Zhejiang Chinese Medical University, Hangzhou, Zhejiang, China

**Keywords:** chronic pelvic pain, hypertonic pelvic floor, pelvic floor dysfunction, postpartum anxiety, urogynecology

## Abstract

Pelvic floor dysfunction (PFD) and postpartum anxiety are commonly assessed and treated separately, although accumulating evidence suggests that psychological distress and pelvic floor muscle over activity may interact. This narrative review synthesizes epidemiological, neurobiological, and rehabilitation evidence and proposes the Anxiety–Hypertonic Pelvic Floor Axis as a framework for this bidirectional association. Postpartum anxiety and chronic stress may contribute to hypothalamic–pituitary–adrenal axis dysregulation, sympathetic predominance, inflammatory signaling, and central sensitization. These processes may contribute to pelvic floor guarding and hypertonicity and may be associated with chronic pelvic pain, dyssynergic defecation, urinary symptoms, and sexual dysfunction. Persistent symptoms may, in turn, intensify anxiety, pain catastrophizing, and fear-avoidance behavior. This framework challenges the assumption that postpartum PFD primarily reflects muscle weakness and that strengthening exercises are universally appropriate. We therefore propose a phenotype-guided, biopsychosocial approach that combines psychological screening with assessment of pelvic floor resting tone and coordination. For women with an anxiety-related hypertonic phenotype, management may integrate cognitive behavioral therapy, mindfulness-based interventions, trauma-informed care, diaphragmatic breathing, reverse Kegels, biofeedback down-training, and manual therapy. Recognition of this potential phenotype may support more individualized clinical assessment and may help avoid indiscriminate strengthening-based treatment. Prospective studies are needed to determine whether phenotype-specific care improves postpartum outcomes.

## 1 Introduction

In the interdisciplinary field of modern obstetrics and rehabilitation medicine, the relationship between postpartum pelvic floor dysfunction (PFD) and mental health is receiving increasing research attention. For a long time, clinical practice in postpartum care has often exhibited a dualistic tendency of “mind–body separation”: obstetricians and physical therapists focus on the repair of muscle structures, the anatomical position of organs, and the functional integrity of sphincters, while psychological services address postpartum depressive and anxiety symptoms. However, a growing body of epidemiological evidence and neurobiological research indicates that these two clinical domains may be linked through complex neural, endocrine, and immune networks. Recently, Shen et al. reported that a multidisciplinary closed-loop care model addressing perinatal anxiety and depression was associated with improved pregnancy outcomes (1), supporting the clinical value of integrated mind–body care. Emerging evidence suggests that psychological distress and pelvic floor dysfunction may interact through overlapping neuroendocrine, autonomic, inflammatory, pain-processing, and behavioral pathways. On this basis, we propose the “Anxiety–Hypertonic Pelvic Floor Axis” as a conceptual, hypothesis-generating framework rather than an established pathological entity.

The traditional biomedical model, when addressing postpartum pelvic issues, has relied predominantly on mechanical muscle strength training, namely Kegel exercises. This approach is based on the underlying assumption that PFD primarily stems from muscle laxity or weakness (hypotonicity). However, this assumption may overlook that some postpartum women can present not only with reduced muscle strength but also with increased muscle tone or impaired relaxation and accompanying neuromuscular dysregulation. Indirect evidence from non-postpartum populations supports the clinical relevance of pelvic floor hypertonicity: in a 2025 study of patients with provoked vestibulodynia, Gao et al. found that the condition was significantly associated with a high-tonus pelvic floor muscle state (2), and Mínguez-Esteban et al. emphasized that an accurate assessment of pelvic floor muscle tone is important for guiding subsequent treatment decisions (3). It has been proposed that, in some patients, such hypertonicity may represent a somatic manifestation of anxiety and a defensive “guarding reflex” in response to perceived threat (4). Recent research further indicates that overactive bladder (OAB) in perinatal women is associated not only with impaired physiological function but also with psychological distress (5).

This review aims to move beyond an exclusively muscle-focused perspective by synthesizing the existing literature into a mechanistic framework of the proposed “Anxiety–Hypertonic Pelvic Floor Axis.” We examine how dysregulation of the hypothalamic–pituitary–adrenal (HPA) axis, inflammatory signaling, and central sensitization mechanisms may contribute to this bidirectional association. A 2026 review by Marano et al. detailed the sex-specific mechanisms of neuroinflammation in female mood disorders and stress vulnerability (6), while a study by Han et al. described how early-life stress may affect key genes of the HPA axis through epigenetic modifications (7). These findings provide biological background for understanding anxiety. Several recent studies also inform the integration of psychological interventions into physical therapy. A randomized controlled trial protocol is currently evaluating a mindfulness-based intervention for postpartum anxiety disorders (8). A 2026 network meta-analysis by Qin et al. reported that online cognitive behavioral therapy (OCBT) ranked favorably for reducing short-term postpartum anxiety symptoms (9). Waqas et al. compared standard CBT with technology-assisted peer-delivered CBT, offering new insights into different forms of psychological intervention for perinatal depression (10). Meanwhile, modern technological approaches are emerging, such as the use of explainable machine learning algorithms by Huang et al. to predict the risk of postpartum depression (11), and a multicenter randomized controlled trial by Zhu et al. that supported a mobile app-based self-management program for urinary incontinence during pregnancy (12). Finally, Motiwala et al. highlighted, from a surgical perspective in patients with endometriosis, the relevance of addressing pelvic floor factors—including hypertonicity—when managing urinary symptoms (13); as this evidence derives from a non-postpartum surgical population, it is considered indirect. Taken together, these strands suggest a potential shift toward a more integrated biopsychosocial approach to postpartum pelvic floor care, while underscoring that direct postpartum evidence remains limited.

A recent preprint has highlighted the broader integration of psychological, social-support, physical, and technology-assisted strategies in postpartum mental-health care (14). Because this work has not yet undergone formal peer review, it is cited here only to provide contemporary context for integrated postpartum rehabilitation and not as evidence validating the proposed Anxiety–Hypertonic Pelvic Floor Axis.

The literature identification and evidence synthesis methods of this narrative review are described in Section 2. The objective of this review is to integrate direct postpartum evidence, indirect clinical evidence, and mechanistic evidence into a coherent, hypothesis-generating framework, rather than to establish causality.

## 2 Methods of literature identification and evidence synthesis

### 2.1 Information sources and search date

A structured narrative literature search was conducted in PubMed/MEDLINE, Scopus, and Web of Science Core Collection, with the final searches performed on 29 August 2026 across all three databases. Additional records were identified through backward and forward citation tracking of key publications.

### 2.2 Search strategy

Search terms combined concepts related to the postpartum period, anxiety or psychological distress, pelvic floor dysfunction or hypertonicity, pelvic pain, autonomic and HPA-axis function, inflammatory signaling, central sensitization, and rehabilitation interventions. Four complementary search strings, translated into the native syntax of each database, were used: a core string addressing postpartum psychological outcomes and pelvic floor dysfunction, and supplementary strings addressing neuroendocrine, inflammatory, autonomic, and central-sensitization mechanisms; rehabilitation interventions; and hypertonic or nonrelaxing pelvic floor states. The full search strings for all three databases, together with the record counts retrieved per string, are provided in Supplementary Table S1.

### 2.3 Eligibility and evidence selection

Eligible publications included peer-reviewed human studies addressing postpartum or perinatal psychological outcomes and pelvic floor symptoms or function, as well as non-postpartum clinical and mechanistic studies when they were relevant to biological plausibility. Publications were excluded when they did not address the population, concepts, or outcomes of interest, when psychological and pelvic floor outcomes were both absent, or when they consisted of commercial or promotional material. Non-peer-reviewed patient-information sources were not used as principal evidence for efficacy, contraindications, or physiological mechanisms. Records were screened by title and abstract, and the full texts of potentially relevant publications were reviewed.

### 2.4 Evidence categorization and narrative synthesis

Evidence was classified as (1) direct postpartum evidence, (2) indirect clinical evidence from non-postpartum pelvic pain or pelvic floor populations, or (3) mechanistic/contextual evidence. This categorization is applied throughout the manuscript to avoid unwarranted extrapolation across populations. The searches returned 14,716 records (PubMed/MEDLINE 3,313; Scopus 6,420; Web of Science Core Collection 4,983); 7,358 records remained after removal of duplicates within and across databases and were screened by title and abstract, and the full texts of potentially relevant publications were reviewed; 56 publications were included in the narrative synthesis. Because this is a narrative rather than a systematic review, no formal meta-analysis or certainty-of-evidence grading was performed; the synthesis is therefore susceptible to selection and interpretive bias, as acknowledged in the Limitations section. Key studies informing the proposed framework are summarized in Table 1.

**Table 1:** Characteristics and relevance of key studies informing the proposed anxiety–hypertonic pelvic floor axis.

Study	Population	Design	Postpartum-specific?	Pelvic floor phenotype/outcome	Psychological outcome	Principal finding	Evidence category
Swenson et al. (15)	Women referred to a specialty postpartum perineal clinic	Retrospective	Yes	Pelvic floor symptoms	Positive EPDS screen	Selected pelvic floor symptoms associated with higher odds of a positive screening result	Direct postpartum
Zhao et al. (16)	80 postpartum women with GAD	Retrospective	Yes	sEMG amplitude, pelvic floor function	GAD-7, HAM-A	Within-group improvements after a 12-week individualized rehabilitation program; no randomized control	Direct postpartum
Wang et al. (5)	Women during the perinatal period	Cross-sectional (latent profile analysis)	Yes (perinatal)	Overactive bladder symptom profiles	Psychological distress	OAB symptom profiles associated with psychological factors	Direct (perinatal)
Glowacka et al. (17)	Pregnant and postpartum women	Prospective cohort	Yes	Genito-pelvic pain	Fear avoidance, depression	Fear avoidance prospectively associated with genito-pelvic pain	Direct postpartum
Joinson et al. (18)	Women, population-based	Prospective cohort	No	Urinary symptoms (incl. SUI, nocturia)	Depression, anxiety	Bidirectional longitudinal associations between urinary symptoms and depression/anxiety	Indirect clinical
Cyr et al. (19)	Females treated for gynecological cancer	Systematic review	No	Bladder, bowel, sexual function; PFM function	Psychological function, QoL	PFMT and education-based therapies showed benefit across multiple domains	Indirect clinical
Bittelbrunn et al. (20)	Women with chronic pelvic pain	Systematic review/meta-analysis	No	CPP; pelvic floor physical therapy outcomes	Mindfulness-related outcomes	PFPT and mindfulness-based approaches associated with improvement	Indirect clinical
Kavitha et al. (21)	Postpartum women with SUI	Randomized clinical trial protocol	Yes	PFM strength, QoL	–	90–90 ball and balloon exercise vs Kegel under evaluation; outcome data not yet available	Direct postpartum (protocol)
Leonardo et al. (22)	Women with stress urinary incontinence	Systematic review/meta-analysis of RCTs	No	SUI; PFMT ± biofeedback	–	PFMT effective for SUI; no consistent added benefit of biofeedback	Indirect clinical
Hagen et al. (23)	Women with urinary incontinence	Multicenter RCT	No	UI; PFMT ± EMG biofeedback	–	No additional benefit of biofeedback-augmented PFMT	Indirect clinical
Lackner et al. (24)	Patients with chronic pelvic pain	Review	No	CPP; CBT components	Catastrophizing, pain	CBT framework and evidence base for chronic pelvic pain	Indirect clinical
Hannibal and Bishop (25)	Pain rehabilitation literature	Review	No	Pain/stress mechanisms	Cortisol/stress	Psychoneuroendocrine rationale for stress management in pain rehabilitation	Mechanistic/contextual
Eller-Smith et al. (26)	Centralized pain literature	Review	No	Centralized pain mechanisms	–	Mechanisms of central sensitization and candidate interventions	Mechanistic/contextual
Afyouni et al. (27)	Nonrelaxing pelvic floor dysfunction literature	Review	No	Urologic manifestations of nonrelaxing PFD	–	Clinical workup and management of nonrelaxing PFD	Mechanistic/contextual (clinical review)

## 3 Epidemiological associations between pelvic floor dysfunction and postpartum mental health

The postpartum period is a window of pronounced physiological and psychological vulnerability in a woman's life. During this time, pelvic floor dysfunction and psychological distress frequently co-occur.

### 3.1 Epidemiological association between pelvic floor dysfunction and mental health

Existing clinical data suggest a substantial burden of psychological symptoms among women with pelvic floor complaints. In a detailed retrospective study of a specialized postpartum perineal clinic, researchers found that among women referred for treatment of pelvic floor symptoms, about 15.6% tested positive on the Edinburgh Postnatal Depression Scale (EPDS) screening (15). This finding indicates a substantial burden of positive psychological screening among women referred for pelvic floor symptoms. Because the study was observational and conducted in a referred specialty population, it does not establish pelvic floor symptoms as a causal trigger of postpartum psychological distress.

Multivariate logistic regression analysis in the same cohort indicated that women reporting urinary incontinence during pregnancy and the postpartum period had higher odds of a positive postpartum depression screening result (aOR 3.81, 95% CI 1.57–9.25, *p* = 0.003) (15). Women referred for pelvic pain also had higher odds of a positive screening result (aOR 2.61) (15). These estimates describe associations with a positive screening result and should not be interpreted as confirmed diagnoses of depression or anxiety disorders.

Furthermore, a systematic review and meta-analysis that included 54 studies and over 630,000 participants reported a substantial prevalence of depression and anxiety among women with different types of PFD: the prevalence of depression was 26.8% in patients with chronic pelvic pain (CPP), 26.3% in patients with urinary incontinence, and 34.9% in patients with pelvic organ prolapse (POP) (28). These findings support consideration of psychological comorbidity when evaluating women with pelvic floor dysfunction, while the direction and mechanisms of the association remain uncertain.

### 3.2 The intersection and overlap of risk factors

When analyzing the epidemiological characteristics of the proposed framework, sociodemographic factors and previous medical history appear to play moderating roles. Studies have shown that a history of depression or anxiety is one of the strongest predictors of a positive psychological screening result in postpartum PFD patients (aOR 2.77) (15). For women who have experienced mental health problems before or during pregnancy, postpartum pelvic floor care may therefore reasonably include attention to psychological well-being.

Moreover, racial differences also appear relevant. Non-Caucasian women had higher odds of a positive postpartum depression screening result (aOR 2.72) (15), a finding that may relate to socioeconomic status, accessibility of medical resources, and cultural factors influencing the expression of pain and emotions. An increased body mass index (BMI) has also been associated with both pelvic floor symptoms and psychological distress in this cohort, possibly because it increases the mechanical load on the pelvic floor and is often linked to metabolic inflammation and psychological stress (15).

### 3.3 Longitudinal evidence suggesting potential bidirectional associations

Regarding the question of temporal sequence, longitudinal studies suggest potential bidirectional associations, although they do not establish causality. A large population-based prospective cohort study showed that anxiety was significantly associated with the subsequent development of nocturia, and that stress urinary incontinence (SUI) was significantly associated with subsequent depressive episodes (hazard ratio 1.37) (18). Evidence from other functional somatic disorders has suggested temporal associations between anxiety and subsequent symptom development; however, these findings are indirect and should not be interpreted as specific evidence for postpartum pelvic floor hypertonicity (29).

Conversely, the persistence of pelvic floor symptoms may, in turn, intensify anxiety and depressive symptoms. Taken together, these observations are compatible with a potential self-reinforcing cycle in which anxiety may contribute to pelvic floor muscle tension and autonomic nervous system dysregulation, while the embarrassment, pain, and reduced quality of life associated with PFD may further aggravate anxiety and depressive symptoms (18, 29). The epidemiological associations between pelvic floor status and psychological outcomes are summarized in Table 2.

**Table 2:** Epidemiological associations between pelvic floor symptoms and psychological outcomes.

Study	Setting	Population	Design	Pelvic floor symptom/predictor	Psychological construct (instrument)	Effect estimate	Interpretation
Swenson et al. (15)	Specialty postpartum perineal clinic, USA	Women referred for postpartum pelvic floor symptoms	Retrospective	Urinary incontinence during pregnancy/postpartum	Positive postpartum depression screen (EPDS)	aOR 3.81 (95% CI 1.57–9.25)	Higher odds of a positive screening result
Swenson et al. (15)	Specialty postpartum perineal clinic, USA	Women referred for postpartum pelvic floor symptoms	Retrospective	Pelvic pain	Positive postpartum depression screen (EPDS)	aOR 2.61	Higher odds of a positive screening result
Swenson et al. (15)	Specialty postpartum perineal clinic, USA	Women referred for postpartum pelvic floor symptoms	Retrospective	History of depression/anxiety (predictor)	Positive postpartum depression screen (EPDS)	aOR 2.77	Prior psychological history associated with positive screening
Peinado Molina et al. (28)	Multiple countries	Women with PFD (mixed populations)	Systematic review/meta-analysis (54 studies)	Chronic pelvic pain	Depression (prevalence)	26.8%	Higher prevalence of depression
Peinado Molina et al. (28)	Multiple countries	Women with PFD (mixed populations)	Systematic review/meta-analysis (54 studies)	Urinary incontinence	Depression (prevalence)	26.3%	Higher prevalence of depression
Peinado Molina et al. (28)	Multiple countries	Women with PFD (mixed populations)	Systematic review/meta-analysis (54 studies)	Pelvic organ prolapse	Depression (prevalence)	34.9%	Higher prevalence of depression
Joinson et al. (18)	Population-based cohort, UK	Women (general population)	Prospective cohort	Stress urinary incontinence	Depressive episodes	HR 1.37	Longitudinal association; not postpartum-specific

## 4 Potential mechanistic links underlying the proposed anxiety–hypertonic pelvic floor axis

To understand how anxiety and increased pelvic floor muscle tone might interact, it is necessary to examine the neuroendocrine, immune, autonomic, central nervous, and musculoskeletal systems. It should be emphasized that much of the evidence cited in this section is mechanistic or derived from non-postpartum populations and therefore provides biological plausibility rather than direct postpartum validation. The proposed Anxiety–Hypertonic Pelvic Floor Axis is summarized in Figure 1, illustrating how postpartum anxiety or stress may initiate multiple psychoneuroimmunological pathways that may contribute to pelvic floor guarding, hypertonicity, functional symptoms, and a potential self-reinforcing cycle of anxiety and fear-avoidance behavior.

**Figure 1:**
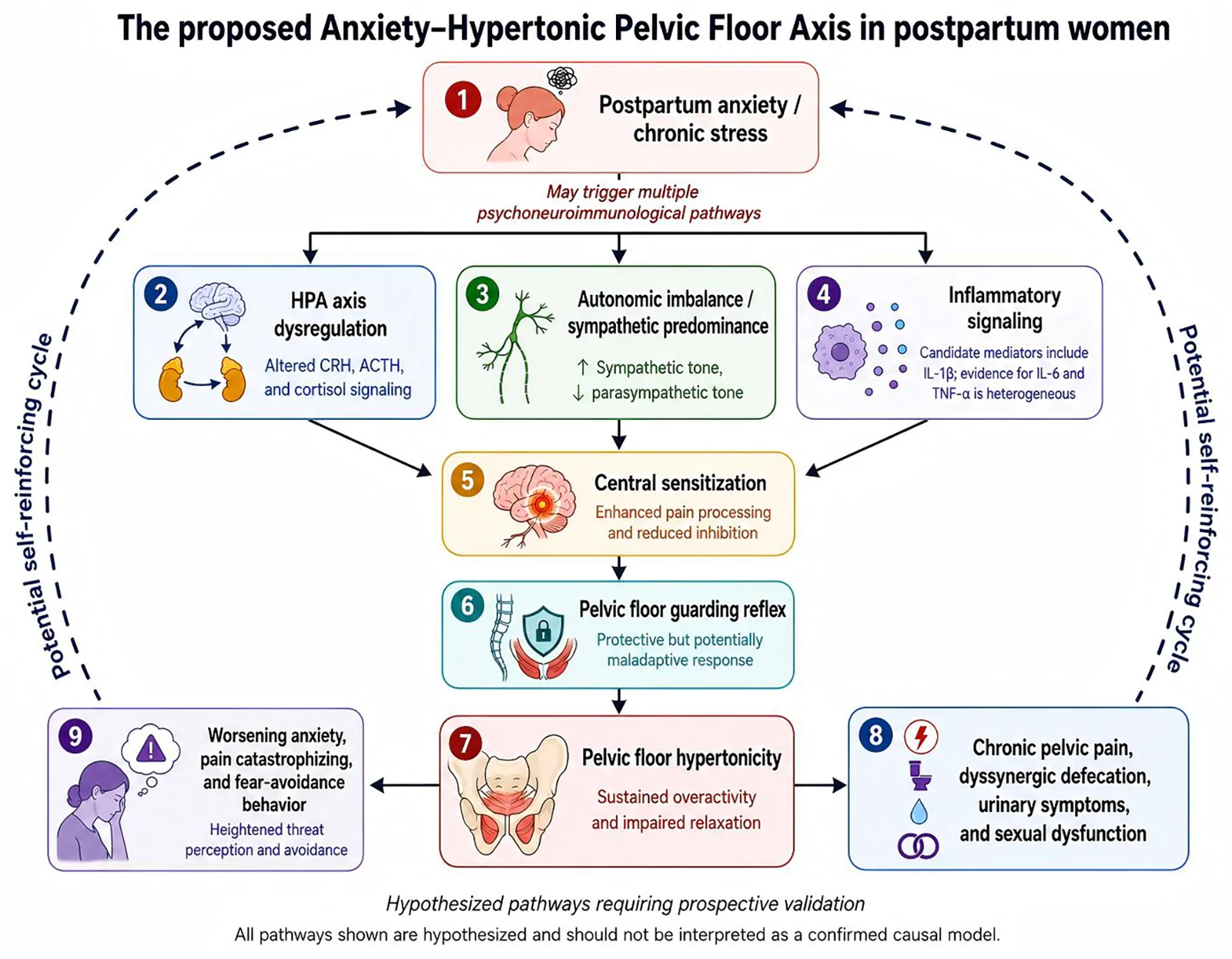
The proposed anxiety–hypertonic pelvic floor axis in postpartum women. Postpartum anxiety and chronic stress may induce hypothalamic–pituitary–adrenal axis dysregulation, autonomic imbalance characterized by sympathetic predominance, and increased inflammatory signaling. These interacting mechanisms may promote central sensitization and a persistent pelvic floor guarding reflex, resulting in pelvic floor hypertonicity. Chronic pelvic pain, dyssynergic defecation, urinary symptoms, and sexual dysfunction may subsequently worsen anxiety and fear-avoidance behavior, thereby potentially forming a self-reinforcing cycle. All pathways depicted are hypothesized and require prospective validation; the figure should not be read as a confirmed causal model.

### 4.1 Neuroendocrine dysregulation: the cascade reaction of the HPA axis and cortisol

The hypothalamic-pituitary-adrenal (HPA) axis is the body's core neuroendocrine system for responding to stress. Under acute stress, activation of the HPA axis leads to the release of cortisol, an adaptive response aimed at mobilizing energy to cope with threats (30). However, in states of postpartum anxiety or chronic stress, the HPA axis may become dysregulated.

This dysfunction is typically manifested as a dysregulation of the cortisol secretion rhythm. Prolonged stress may lead to persistently elevated cortisol levels, which in turn may have neurotoxic effects on the hippocampus and prefrontal cortex through glucocorticoid receptors (GR), weakening the brain's ability to regulate emotions (31). More complexly, in centralized pain syndromes such as chronic pelvic pain (CPP), a state of “hypocortisolism” or blunting of the HPA axis is often observed (26). This low cortisol state may impair the effective suppression of inflammatory responses, leading to elevated systemic inflammation levels, which in turn may sensitize peripheral pain receptors.

The metabolic effects of cortisol, in synergy with the sympathetic nervous system (SNS), maintain a “fight-or-flight” physiological state (31). In this state, overall muscle tension increases in preparation for action. The pelvic floor muscles, as part of the core stabilizing muscle group, are particularly sensitive to this sympathetic activation. Sustained sympathetic predominance may maintain the pelvic floor muscles in a state of heightened tension, contributing to what has been described as nonrelaxing pelvic floor dysfunction (27).

### 4.2 Inflammatory signaling as a potential mechanistic link

Research in psychoneuroimmunology (PNI) suggests that inflammatory signaling may be one of the links connecting emotional disorders and somatic pain. Depression and anxiety have themselves been associated with pro-inflammatory states (32).

In postpartum women, depressive symptoms have been investigated in relation to several inflammatory mediators, particularly IL-1β, IL-6, and TNF-β (33). A recent systematic review found the most consistent evidence for elevated IL-1β, whereas findings for IL-6, IL-10, and TNF-β were heterogeneous (34). In women with chronic pelvic pain, IL-6 levels have been positively correlated with pain severity (35), and dynamic changes in serum IL-6, IL-10, and TNF-β have also been reported during postpartum perineal edema rehabilitation, indicating active postpartum inflammatory regulation (36). Mechanistically, the combined action of various cytokines may contribute to “hyperalgesic priming,” rendering local tissues in the pelvic floor (such as minor injuries from childbirth) abnormally sensitive to mechanical stimuli. Even after the tissue has healed, the “memory” left by these inflammatory mediators may maintain the transmission of pain signals, potentially contributing to chronic pelvic pain (26). Overall, inflammatory signaling should be regarded as a plausible contributing mechanism rather than a confirmed driver of postpartum pelvic floor hypertonicity.

### 4.3 Autonomic nervous system and vagal regulation

The vagus nerve is the main component of the parasympathetic nervous system, responsible for the body's “rest and digest” functions, and has anti-inflammatory effects. In a healthy physiological state, the vagus nerve buffers the stress response by regulating heart rate variability (HRV), a mechanism described within polyvagal theory as the “vagal brake” (37).

Patients with anxiety typically exhibit reduced vagal tone, with sympathetic predominance. This disruption of autonomic balance may influence pelvic floor function. The pelvic floor muscles and the diaphragm are functionally and anatomically coordinated: during inhalation, the diaphragm descends and the pelvic floor muscles lengthen; during exhalation, the diaphragm ascends, and the pelvic floor muscles return toward their resting position (38). The breathing pattern associated with anxiety is usually shallow and rapid chest breathing, which reduces the range of motion of the diaphragm. This may deprive the pelvic floor muscles of the physiological signal for rhythmic relaxation with breathing, favoring sustained contraction (39, 40).

### 4.4 The defense mechanism of the musculoskeletal system: guarding reflex and hypertonicity

The “guarding reflex” is a core pathophysiological concept used to explain pelvic floor hypertonicity. It is an instinctive protective response in which the pelvic floor muscles contract involuntarily to protect the pelvic organs when the body perceives pain, threat, or uncertainty (27).

In the context of postpartum anxiety, this reflex may be excessively and continuously activated. Anxiety itself is a continuous anticipation of a “potential threat.” The amygdala constantly sends alarm signals to the brainstem, increasing the activity of gamma motor neurons through the reticulospinal tract. This may lead to increased sensitivity of the muscle spindles, maintaining a state of high muscle tension (30). This state, known as “chronic guarding,” has been associated not only with muscle fatigue and ischemic pain but also with impaired urination, defecation, and sexual function (4, 27).

### 4.5 Central sensitization and the persistence of pain

Long-term peripheral inflammatory input and persistent psychological stress may eventually lead to plastic changes in the central nervous system, namely central sensitization. At this stage, the threshold of neurons in the dorsal horn of the spinal cord is lowered, causing a painful response (allodynia) to normally non-painful stimuli such as bladder filling, rectal distension, or sexual contact (26).

Central sensitization is one of several mechanisms proposed to contribute to the persistence and amplification of chronic pelvic pain (26). Patients often report widespread pain, fatigue, sleep disturbances, and hypersensitivity to light and sound, all of which are manifestations of an amplified gain in the central nervous system (26). At this point, the problem is no longer confined to the pelvic floor itself but has become part of a systemic neurological dysregulation. Anxiety may further amplify pain signals through cognitive “catastrophizing,” potentially forming a self-reinforcing cycle (17).

## 5 Clinical manifestations and diagnostic challenges

The proposed framework may be clinically relevant to a subset of postpartum women presenting with overlapping pelvic floor symptoms, pain, and psychological distress, whose symptoms are sometimes attributed solely to simple organ lesions or infections.

### 5.1 Paradoxical puborectalis contraction and dyssynergic defecation

Dyssynergic defecation (also known as anismus) is one of the most typical functional disorders of a hypertonic pelvic floor. During normal defecation, the puborectalis muscle should relax to open the anorectal angle. In dyssynergic defecation, the puborectalis muscle and/or external anal sphincter may fail to relax appropriately or may contract paradoxically during attempted defecation (41).

Psychological comorbidity, including anxiety and depression, is common in patients with evacuation disorders (42), although a direct causal role of anxiety in generating dyssynergia has not been established. Patients may also develop defecation avoidance behavior because of pain or embarrassment, potentially exacerbating constipation and pelvic floor dysfunction.

### 5.2 Chronic pelvic pain and myofascial trigger points

Hypertonic muscles, being in a shortened state for a long time, are prone to forming myofascial trigger points (TrPs). These points not only cause local tenderness but also produce referred pain, mimicking the symptoms of gynecological or urological diseases (43). For example, trigger points in the obturator internus muscle can cause symptoms similar to sciatica, while spasms of the levator ani muscle may manifest as deep vaginal or rectal pain.

In postpartum women, pelvic pain may have multiple overlapping causes, including tissue injury, infection, musculoskeletal dysfunction, and pain sensitization. Hypertonic or myofascial contributors should therefore be considered as part of a differential assessment rather than assumed to be the primary cause.

### 5.3 Postpartum sexual dysfunction and psychological avoidance

Dyspareunia and vaginismus are other common manifestations of a hypertonic pelvic floor. Postpartum women, due to fear of wound dehiscence or anxiety about their body image, may experience intense anticipatory anxiety during sexual contact (17). This anxiety may instantly trigger a defensive contraction of the pelvic floor muscles, leading to pain during intercourse.

According to the “Fear-Avoidance Model,” the experience of pain leads to a fear of sexual activity, which in turn results in avoidance behavior. Persistent fear and avoidance may reinforce pain-related distress, reduce sexual activity and confidence, and contribute to ongoing sexual dysfunction; whether avoidance itself increases postpartum pelvic floor resting tone remains uncertain (17).

### 5.4 Limitations and innovations in diagnostic tools: sEMG and high-resolution manometry

While the traditional digital rectal examination is important, it is highly subjective. Surface electromyography (sEMG) and high-resolution anorectal manometry (HRAM) provide more objective assessment tools.

In patients with the “anxiety-hypertonic” profile, sEMG typically shows an elevated baseline resting tone and an inability to quickly return to baseline after attempting to relax (relaxation deficit) (27). Zhao et al. reported impaired pelvic floor function based on sEMG-derived functional measures in postpartum women with GAD; however, the study did not establish objectively elevated pelvic floor resting tone or confirm a hypertonic phenotype (16). This subtle distinction is key to formulating a treatment plan. Prescribing strengthening exercises solely on the basis of reduced contraction amplitude, without assessing resting tone and relaxation capacity, may be inappropriate in patients with a nonrelaxing or hypertonic phenotype.

## 6 Limitations of non-individualized rehabilitation models

Faced with postpartum pelvic floor dysfunction, commonly used treatment plans are often predominantly muscle-centered and may be inappropriate for specific subgroups of patients.

### 6.1 Limitations of non-individualized strengthening in hypertonic or nonrelaxing pelvic floor dysfunction

Since their introduction in 1948, Kegel exercises have almost become synonymous with pelvic floor rehabilitation (44). Their core logic is to enhance muscle strength through repeated active contractions to combat urinary incontinence and organ prolapse. Pelvic floor muscle strengthening is effective for several forms of postpartum pelvic floor dysfunction, particularly stress urinary incontinence (22). However, strengthening-focused exercise may be inappropriate or poorly tolerated in some women with objectively confirmed nonrelaxing or hypertonic pelvic floor dysfunction (4, 45). When the pelvic floor muscles are already in a state of spasm, shortening, and overactivity, further strengthening contractions may aggravate muscle ischemia, hypoxia, and pain (45).

Furthermore, correct performance of Kegel exercises is not trivial. In a classic study, only 49% of women produced an ideal Kegel contraction after brief verbal instruction, and 25% used a technique—such as Valsalva—that could potentially promote incontinence (46). Common errors include breath-holding, contraction of the gluteus maximus or inner thigh muscles, or bearing down, which increase intra-abdominal pressure and may be counterproductive (45, 46).

### 6.2 Issues of adherence and recurrence with singular physical therapy

Coexisting anxiety, pain catastrophizing, and fear of symptom exacerbation may interfere with engagement in rehabilitation in some patients (17). However, direct evidence that these psychological factors independently determine adherence or recurrence in postpartum pelvic floor rehabilitation remains limited.

Although pelvic floor rehabilitation can improve selected postpartum pelvic floor outcomes, long-term evidence remains heterogeneous, and relatively few studies have evaluated sustained outcomes beyond six months (47). Whether the addition of psychological support improves long-term adherence or reduces recurrence specifically in postpartum women with hypertonic pelvic floor dysfunction remains uncertain. In a retrospective study of postpartum women with GAD, a comprehensive rehabilitation program integrating psychological support reported a session adherence rate of 91.5% and no serious adverse events (16); however, given its retrospective, nonrandomized design, these findings should be interpreted as preliminary.

### 6.3 The clinical consequences of neglecting psychological factors

Neglecting the psychological dimension of the proposed framework may contribute to a “revolving door” phenomenon in clinical practice, in which patients repeatedly seek medical help but their symptoms persist. Unrecognized anxiety and depression have been associated with poorer treatment outcomes for lower urinary tract symptoms (LUTS) (48). If clinicians focus only on the local bladder or muscles without considering the patient's psychological state, treatment may fail to address relevant psychological and behavioral contributors.

## 7 Integrated rehabilitation strategies: a biopsychosocial perspective

A biopsychosocial perspective may complement conventional postpartum pelvic floor rehabilitation by considering psychological distress, pain processing, autonomic factors, and behavioral responses alongside tissue healing and muscle function. A proposed clinical pathway for the integrated assessment and rehabilitation of postpartum women with anxiety-related pelvic floor dysfunction is presented in Figure 2. The pathway emphasizes sequential psychological and pelvic floor screening, phenotype classification, objective functional assessment, multidisciplinary intervention, and longitudinal outcome monitoring. This pathway is a research-informed framework that requires prospective validation and should not be interpreted as an established treatment algorithm.

**Figure 2:**
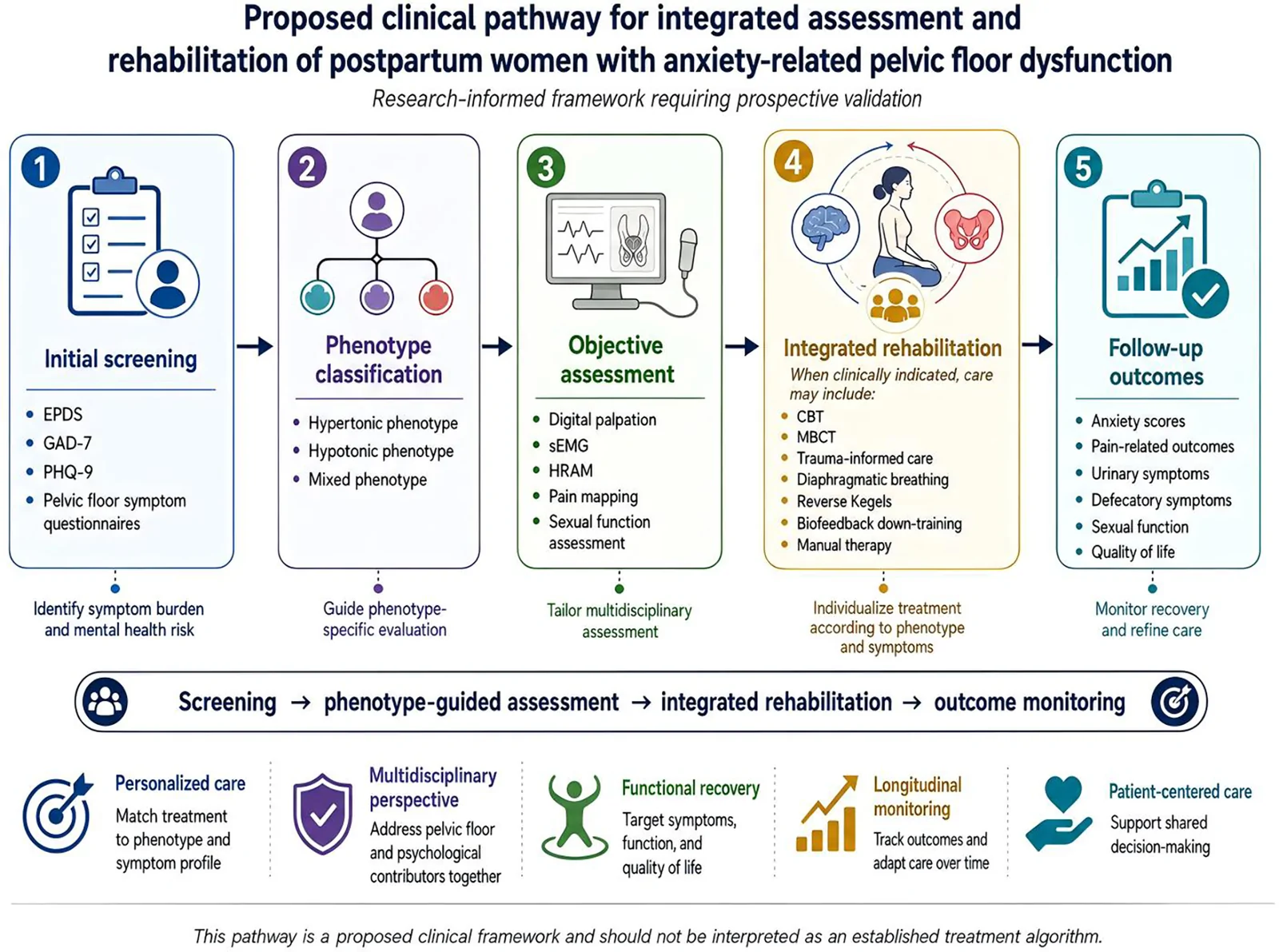
Proposed clinical pathway for integrated assessment and rehabilitation of postpartum women with anxiety-related pelvic floor dysfunction. Initial assessment includes screening for postpartum anxiety, depression, and pelvic floor symptoms using instruments such as the EPDS, GAD-7, PHQ-9, and pelvic floor symptom questionnaires. Patients are subsequently classified as having a hypertonic, hypotonic, or mixed pelvic floor phenotype and undergo phenotype-guided objective assessment. For patients with anxiety-related pelvic floor hypertonicity, integrated rehabilitation may include CBT, MBCT, trauma-informed care, diaphragmatic breathing, reverse Kegels, biofeedback down-training, and manual therapy. Treatment response should be evaluated using psychological, pain-related, urinary, defecatory, sexual-function, and quality-of-life outcomes.

### 7.1 The role of psychological intervention in physical therapy

Psychological assessment and intervention may be appropriate early in care for women with clinically significant anxiety, depressive symptoms, pain catastrophizing, or fear-avoidance, rather than being reserved only for refractory symptoms. Indirect evidence from non-postpartum populations—including gynecological cancer survivors with pelvic floor dysfunction—suggests that multimodal physical therapy combined with psychological support may improve dyspareunia and quality of life (19). The main intervention goals are, firstly, to reduce excessive arousal levels by supporting regulation of the HPA axis and the autonomic nervous system; secondly, to interrupt the fear-avoidance cycle caused by pain by reshaping the patient's cognitive perception of pain to reduce catastrophic thinking; and finally, to enhance the patient's sense of self-efficacy, allowing them to feel in control of their symptoms rather than being controlled by them.

### 7.2 Cognitive behavioral therapy (CBT) and mindfulness-based interventions

CBT- and mindfulness-based approaches have supportive evidence for selected psychological and chronic pain outcomes, although direct evidence for treating postpartum pelvic floor hypertonicity remains limited. Within the CBT framework, key components include psychoeducation for the patient, explaining the concept that “hurt does not equal harm,” and clarifying how anxiety may contribute to muscle tension (24). The therapist guides the patient through cognitive restructuring, identifying and challenging automatic negative thoughts such as “I will never get better,” and replacing them with more realistic coping thoughts (24). At the same time, through activity pacing, patients are guided to gradually increase their activity levels within a pain-tolerant range, aiming to avoid disuse from excessive rest and to prevent symptom flare-ups from over-activity (24). In mindfulness-based protocols, the body scan is a key technique. It guides the patient to gently bring attention to the pelvic floor area, observing sensations without judgment. This may help distinguish between “tension” and “pain” and interrupt automatic reactive contraction to discomfort (49). Acceptance-based strategies encourage patients to reduce the struggle against discomfort rather than “fighting” it by tensing their muscles; acceptance- and mindfulness-based interventions have shown benefits for chronic pain in meta-analyses (50), although these data derive predominantly from non-postpartum chronic pain populations.

### 7.3 Trauma-informed care (TIC): principles and practice

Given that a substantial proportion of patients seeking pelvic health care report a history of sexual trauma (51), trauma-informed principles may be particularly relevant to pelvic floor assessment and rehabilitation, because intimate examination and treatment can be distressing for some patients, especially those with a history of trauma. Postpartum-specific outcome evidence remains limited. The core of TIC is not to treat the trauma itself (that is the role of a psychotherapist) but to create a safe and respectful treatment environment that avoids re-traumatization (51, 52). In pelvic rehabilitation, the practice of TIC follows several key principles. The first is to ensure physical and emotional safety—for example, arranging the treatment room to avoid a sense of confinement and allowing patients to keep some clothing on during certain examinations to increase their sense of security. The second is informed consent: before any physical contact, especially internal examinations, explicit verbal consent should be obtained from the patient, who should be clearly informed of the right to pause or stop at any time. Third, empowerment is crucial, which means involving the patient actively in treatment decisions, transforming them from a passive recipient to an active collaborator. Finally, where appropriate, sensitive screening can help identify potential trauma history and anxiety levels, allowing timely adjustment of the treatment pace (51–53).

### 7.4 Relaxation-oriented physical therapy: reverse Kegels and diaphragmatic breathing training

For the hypertonic pelvic floor, the focus of physical therapy generally shifts from “strengthening” to “relaxing” and “lengthening” (4). Among the available techniques, diaphragmatic breathing is one commonly used relaxation-oriented technique that may support pelvic floor lengthening and autonomic regulation. Through deep inhalation, the diaphragm descends, and intra-abdominal pressure moderately increases, thereby promoting eccentric lengthening of the pelvic floor muscles; slow breathing has also been associated with increased vagal activity and reduced autonomic arousal (38, 40, 54). The reverse Kegel, which is the opposite of the traditional Kegel exercise, instructs the patient to consciously “let go” or “release” the pelvic floor, with the aim of reducing elevated resting tone (4, 55). In addition, the 90–90 ball and balloon exercise, as an advanced training method, requires the patient to blow into a balloon in a specific position (supine with hips and knees flexed at 90 degrees), an action intended to coordinate the deep core muscles with pelvic floor relaxation. The 90–90 ball and balloon exercise is being evaluated against conventional Kegel exercise in postpartum women with stress urinary incontinence. Because the cited publication is a randomized clinical trial protocol, comparative outcome data were not yet available in that report (21). Its effectiveness, particularly in women with objectively confirmed hypertonic pelvic floor dysfunction, therefore remains uncertain.

### 7.5 Biofeedback and multidisciplinary collaboration model

Biofeedback utilizes sEMG or pressure probes to transform the invisible activity of the pelvic floor muscles into a visual signal. For patients with hypertonicity, the main approach is “down-training,” which involves setting low threshold targets to reward the relaxed state of the muscles (4, 27).

When clinically indicated and where relevant expertise is available, rehabilitation may be delivered within a multidisciplinary framework involving pelvic health therapists, psychological professionals, and obstetric or gynecological clinicians. The physical therapist is responsible for retraining muscle function, the psychologist for anxiety management, and the obstetrician/gynecologist for ruling out organic pathologies. All parties communicate regularly, sharing the patient's functional measurements and psychological scale scores to dynamically adjust the treatment plan (16).

## 8 Current clinical evidence and evidence gaps

Direct evidence evaluating integrated psychological and pelvic floor rehabilitation in postpartum women is limited, while indirect evidence from chronic pelvic pain and other pelvic floor populations provides supportive but non-specific context.

### 8.1 Direct postpartum evidence for integrated rehabilitation and its limitations

Zhao et al. retrospectively evaluated 80 postpartum women with generalized anxiety disorder who completed a 12-week individualized pelvic floor rehabilitation program combining supervised pelvic floor muscle training, biofeedback, relaxation techniques, and psychological support (16). Within-group improvements were reported for psychological outcomes—GAD-7 scores decreased from 14.8 ± 3.2 at baseline to 8.2 ± 2.9 at week 12 (*P* < 0.001), and HAM-A scores from 22.6 ± 4.5 to 12.4 ± 3.8 (*P* < 0.001)—as well as for pelvic floor function, with sEMG amplitude increasing from 22.4 ± 5.6 *μ*V to 35.9 ± 6.8 μV (*P* < 0.001), and for quality of life, with WHOQOL-BREF psychological and physical domain scores improving by 28.5% and 25.8%, respectively; program adherence was 91.5% and no serious adverse events were reported (16). These findings provide preliminary clinical evidence for the potential value of integrated rehabilitation in postpartum women with GAD. However, the retrospective design and absence of randomized allocation preclude causal inference regarding treatment efficacy, and the observed improvements cannot be attributed to specific components of the multicomponent intervention (16).

Indirect clinical evidence from non-postpartum populations provides additional context. A systematic review and meta-analysis of women with chronic pelvic pain reported that pelvic floor physical therapy and mindfulness-based approaches were associated with improvements in sexual function and related outcomes (20). A case report described improvement of persistent genital arousal disorder/genito-pelvic dysesthesia—a condition associated with pelvic floor overactivity—following pelvic floor physical therapy (56). These findings provide mechanistic or clinical plausibility but should not be interpreted as direct evidence in postpartum women.

### 8.2 Insights from long-term follow-up data

With respect to long-term efficacy, a large multicenter randomized trial found no additional benefit of adding electromyographic biofeedback to pelvic floor muscle training for urinary incontinence (23); it should be noted, however, that in such designs biofeedback is typically used to augment strengthening rather than relaxation-oriented down-training.

Conversely, indirect evidence from studies addressing erroneous movement patterns and psychological-behavioral factors suggests more durable benefits. For dyssynergic defecation, controlled studies and long-term follow-up data indicate that biofeedback therapy is more effective than laxatives and is not associated with significant adverse effects (41); this evidence derives from non-postpartum populations with defecatory disorders and should be extrapolated to postpartum pelvic floor hypertonicity only with caution. These observations are consistent with the concept that restoring coordinated neuromuscular control may be as important as exercising the muscles themselves.

## 9 Discussion

This narrative review integrates epidemiological, mechanistic, and rehabilitation literature to propose the Anxiety–Hypertonic Pelvic Floor Axis as a conceptual framework linking postpartum psychological distress with pelvic floor hypertonicity. Its principal contribution is to bring psychological distress, pelvic floor muscle tone, pain processing, and functional symptoms into a single hypothesis-generating model.

Direct postpartum evidence remains limited. A retrospective study of a specialty postpartum perineal clinic documented associations between pelvic floor symptoms and a positive postpartum depression screening result (15), and a retrospective study of 80 postpartum women with GAD reported within-group improvements following a 12-week individualized rehabilitation program (16). Both studies are observational, and neither can establish causality or treatment efficacy.

Much of the supporting literature is indirect, deriving from women with chronic pelvic pain, provoked vestibulodynia, dyssynergic defecation, endometriosis, or gynecological cancer, or from mechanistic studies of the HPA axis, autonomic function, inflammatory signaling, and central sensitization. These data provide biological plausibility but should not be interpreted as direct evidence in postpartum women.

Clinically, the framework supports individualized assessment of psychological symptoms and pelvic floor phenotype before selecting interventions. In women with objectively confirmed hypertonicity or impaired pelvic floor relaxation, early rehabilitation may reasonably prioritize down-training, breathing, pain management, and relaxation-oriented strategies before progressive strengthening is considered; conversely, strengthening remains appropriate for women with weakness-predominant presentations. The framework does not justify universal prohibition of any single modality, nor does it currently support a standardized treatment algorithm. When clinically indicated, psychological interventions and relaxation-oriented physical therapies may be considered within an individualized, phenotype-guided multidisciplinary strategy; their comparative effectiveness and optimal sequencing in postpartum women with objectively confirmed hypertonicity require prospective evaluation.

Future research should prioritize prospective longitudinal studies to clarify temporal relationships, objective measurement of pelvic floor tone, mediation analyses of candidate mechanisms, and phenotype-stratified randomized trials of integrated interventions. Exploratory biomarkers—including inflammatory profiles such as IL-6 and TNF-β and the cortisol awakening response (CAR)—have been proposed as research hypotheses for objectively assessing inflammatory and stress states (25, 32), but their clinical utility in guiding postpartum pelvic floor care remains untested. The proposed framework may help organize future research on the interaction between postpartum psychological distress and pelvic floor dysfunction, but it remains insufficiently validated for routine clinical decision-making.

## 10 Limitations of the current evidence and review

This review has several limitations. First, it is a narrative rather than systematic review and is therefore susceptible to literature-selection and interpretive bias despite the use of a structured search strategy. Second, direct postpartum evidence simultaneously assessing anxiety and objectively measured pelvic floor hypertonicity remains limited. Third, the included literature is heterogeneous with respect to populations, pelvic floor phenotypes, psychological constructs, and outcome measures. Fourth, several mechanistic and therapeutic inferences are derived from non-postpartum populations, including women with chronic pelvic pain, vestibulodynia, endometriosis, or other pelvic floor disorders; these findings should be considered indirect. Fifth, integrated rehabilitation studies often use multicomponent interventions and nonrandomized designs, limiting attribution of observed improvements to specific treatment elements. Finally, the proposed Anxiety–Hypertonic Pelvic Floor Axis has not been prospectively validated as a unified causal pathway. It should therefore be regarded as a hypothesis-generating framework that requires longitudinal and phenotype-stratified prospective testing.

## 11 Conclusion

Available evidence suggests an association between postpartum psychological distress and pelvic floor dysfunction, while neuroendocrine, autonomic, inflammatory, pain-processing, and behavioral mechanisms provide biological plausibility for reciprocal interactions. However, direct evidence specifically linking postpartum anxiety to objectively measured pelvic floor hypertonicity remains limited.

We therefore propose the Anxiety–Hypertonic Pelvic Floor Axis as a hypothesis-generating framework rather than an established pathological entity. Clinically, this framework supports individualized assessment of psychological symptoms and pelvic floor phenotype before selecting strengthening- or relaxation-oriented interventions. Prospective longitudinal studies and phenotype-stratified intervention trials are required before the framework can be regarded as a validated causal model or treatment pathway.
