# Supplementary material for: A proposed anxiety–hypertonic pelvic floor axis in postpartum women: a narrative review of mechanistic links and rehabilitation implications
Source: Front Surg. 2026 Sep 24;13:1929266. doi: 10.3389/fsurg.2026.1929266 (PMC13646942; doi:10.3389/fsurg.2026.1929266)
Supplement: Supplementary file 1 [file Table1.docx]

**Supplementary Material**

Supplementary Table S1. Full search strings and record counts by database (PubMed/MEDLINE, Scopus, and Web of Science Core Collection; final search date 29 August 2026).

| **Database** | **Search** | **Focus** | **Full search string** | **Date run** | **Records returned** |
| --- | --- | --- | --- | --- | --- |
| PubMed/MEDLINE | S1 | Postpartum psychological outcomes × pelvic floor dysfunction | (("postpartum period"[MeSH Terms] OR postpartum[tiab] OR postnatal[tiab] OR perinatal[tiab]) AND ("pelvic floor disorders"[MeSH Terms] OR "pelvic floor"[tiab] OR hypertonic*[tiab] OR "pelvic pain"[tiab]) AND ("anxiety"[MeSH Terms] OR anxiety[tiab] OR "depression"[MeSH Terms] OR depress*[tiab] OR "psychological distress"[tiab])) | 29 Aug 2026 | 93 |
|  | S2 | Neuroendocrine, inflammatory, autonomic and central-sensitization mechanisms | ((postpartum[tiab] OR postnatal[tiab] OR perinatal[tiab]) AND ("HPA axis"[tiab] OR cortisol[tiab] OR inflammation[tiab] OR cytokine*[tiab] OR "central sensitization"[tiab] OR autonomic[tiab]) AND (depress*[tiab] OR anxi*[tiab])) | 29 Aug 2026 | 1,476 |
|  | S3 | Rehabilitation interventions | (("pelvic floor"[tiab] OR "pelvic pain"[tiab]) AND ("physical therapy"[tiab] OR physiotherap*[tiab] OR biofeedback[tiab] OR "pelvic floor muscle training"[tiab] OR mindfulness[tiab] OR "cognitive behavioral"[tiab]) AND (random*[tiab] OR trial[tiab] OR "systematic review"[tiab] OR meta-analysis[pt])) | 29 Aug 2026 | 1,674 |
|  | S4 | Hypertonic / nonrelaxing pelvic floor states | ("nonrelaxing pelvic floor"[tiab] OR "non-relaxing pelvic floor"[tiab] OR "hypertonic pelvic floor"[tiab] OR "overactive pelvic floor"[tiab] OR "pelvic floor hypertonicity"[tiab]) | 29 Aug 2026 | 70 |
| Scopus | S1 | Postpartum psychological outcomes × pelvic floor dysfunction | TITLE-ABS-KEY( ( postpartum OR postnatal OR perinatal ) AND ( "pelvic floor" OR "pelvic floor disorder*" OR hypertonic* OR "pelvic pain" ) AND ( anxiety OR depress* OR "psychological distress" ) ) | 29 Aug 2026 | 216 |
|  | S2 | Neuroendocrine, inflammatory, autonomic and central-sensitization mechanisms | TITLE-ABS-KEY( ( postpartum OR postnatal OR perinatal ) AND ( "HPA axis" OR cortisol OR inflammation OR cytokine* OR "central sensitization" OR autonomic ) AND ( depress* OR anxi* ) ) | 29 Aug 2026 | 2,482 |
|  | S3 | Rehabilitation interventions | TITLE-ABS-KEY( ( "pelvic floor" OR "pelvic pain" ) AND ( "physical therapy" OR physiotherap* OR biofeedback OR "pelvic floor muscle training" OR mindfulness OR "cognitive behavioral" ) AND ( random* OR trial OR "systematic review" OR "meta-analysis" ) ) | 29 Aug 2026 | 3,615 |
|  | S4 | Hypertonic / nonrelaxing pelvic floor states | TITLE-ABS-KEY( "nonrelaxing pelvic floor" OR "non-relaxing pelvic floor" OR "hypertonic pelvic floor" OR "overactive pelvic floor" OR "pelvic floor hypertonicity" ) | 29 Aug 2026 | 107 |
| Web of Science Core Collection | S1 | Postpartum psychological outcomes × pelvic floor dysfunction | TS=( ( postpartum OR postnatal OR perinatal ) AND ( "pelvic floor" OR "pelvic floor disorder*" OR hypertonic* OR "pelvic pain" ) AND ( anxiety OR depress* OR "psychological distress" ) ) | 29 Aug 2026 | 141 |
|  | S2 | Neuroendocrine, inflammatory, autonomic and central-sensitization mechanisms | TS=( ( postpartum OR postnatal OR perinatal ) AND ( "HPA axis" OR cortisol OR inflammation OR cytokine* OR "central sensitization" OR autonomic ) AND ( depress* OR anxi* ) ) | 29 Aug 2026 | 2,397 |
|  | S3 | Rehabilitation interventions | TS=( ( "pelvic floor" OR "pelvic pain" ) AND ( "physical therapy" OR physiotherap* OR biofeedback OR "pelvic floor muscle training" OR mindfulness OR "cognitive behavioral" ) AND ( random* OR trial OR "systematic review" OR "meta-analysis" ) ) | 29 Aug 2026 | 2,352 |
|  | S4 | Hypertonic / nonrelaxing pelvic floor states | TS=( "nonrelaxing pelvic floor" OR "non-relaxing pelvic floor" OR "hypertonic pelvic floor" OR "overactive pelvic floor" OR "pelvic floor hypertonicity" ) | 29 Aug 2026 | 93 |
| Total (all databases) |  |  |  |  | 14,716 (3,313 PubMed/MEDLINE; 6,420 Scopus; 4,983 Web of Science) |
